# The Stearoyl-CoA Desaturase-1 (Desat1) in *Drosophila* cooperated with Myc to Induce Autophagy and Growth, a Potential New Link to Tumor Survival

**DOI:** 10.3390/genes8050131

**Published:** 2017-04-28

**Authors:** Chiara Paiardi, Zhasmine Mirzoyan, Sheri Zola, Federica Parisi, Andrea Vingiani, Maria Enrica Pasini, Paola Bellosta

**Affiliations:** 1 Department of Biosciences, University of Milan, Via Celoria 26, 20133 Milan, Italy; chiara.paiardi@unimi.it (C.P.); zhasmine.mirzoyan@unimi.it (Z.M.); maria.pasini@unimi.it (M.E.P.); 2 Center of Integrated Biology (CiBio), University of Trento, Via Sommarive 9, 38123 Trento, Italy; sherizola@gmail.com; 3 Crown Bioscience Inc., Santa Clara, CA 950524, USA; fparisi@crownbio.com; 4 Department of Pathology, European Institute of Oncology (IEO), Via Ripamonti 435, 20100 Milan, Italy; andrea.vingiani@unimi.it

**Keywords:** Myc, SCD-1/Desat1, autophagy, growth, lipid metabolism, prostate tumors, *Drosophila*

## Abstract

Lipids are an important energy supply in our cells and can be stored or used to produce macromolecules during lipogenesis when cells experience nutrient starvation. Our proteomic analysis reveals that the *Drosophila* homologue of human Stearoyl-CoA desaturase-1 (Desat1) is an indirect target of Myc in fat cells. Stearoyl-CoA desaturases are key enzymes in the synthesis of monounsaturated fatty acids critical for the formation of complex lipids such as triglycerides and phospholipids. Their function is fundamental for cellular physiology, however in tumors, overexpression of SCD-1 and SCD-5 has been found frequently associated with a poor prognosis. Another gene that is often upregulated in tumors is the proto-oncogene *c-myc*, where its overexpression or increased protein stability, favor cellular growth. Here, we report a potential link between Myc and Desat1 to control autophagy and growth. Using *Drosophila*, we found that expression of Desat1, in metabolic tissues like the fat body, in the gut and in epithelial cells, is necessary for Myc function to induce autophagy a cell eating mechanism important for energy production. In addition, we observed that reduction of Desat1 affects Myc ability to induce growth in epithelial cells. Our data also identify, in prostatic tumor cells, a significant correlation between the expression of Myc and SCD-1 proteins, suggesting the existence of a potential functional relationship between the activities of these proteins in sustaining tumor progression.

## 1. Introduction

Cancer cells require an extensive supply of energy to grow, and when they are constrained by limited nutrients, they can reprogram metabolic pathways to allow for continued growth. One of the genes that masters these metabolic switches is the proto-oncogene *c-myc* [1].

Myc function is highly conserved between humans and flies and in *Drosophila* controls fundamental cellular processes including growth, proliferation, and apoptosis [2]. *Drosophila* Myc activity is downstream of nutrient and growth factor signaling, including RAS and the insulin/TOR signaling pathways that control its protein stability [3,4,5]. Moreover, in the fat body, a metabolic tissue with similar physiological functions to mammalian adipose tissue and liver [6], *myc-mRNA* is transcriptionally induced by FOXO during starvation, suggesting a function for Myc in controlling animal survival [7,8].

Myc overexpression during starvation activates a switch in cellular metabolism, triggering glycolytic flux and enhancing metabolic pathways, including glycolysis and alternative routes like glutamine signaling [7]. This function of Myc is conserved in human and *Drosophila* cells, where it was shown to induce metabolic changes allowing cells of the wing imaginal discs to acquire a competitive behavior (winner cells) over wild-type loser cells in the presence of a fully activated p53 [9]. This behavior was also demonstrated during the initial steps of tumorigenesis, when the overexpression of tumor-promoting genes (i.e., *c-myc*) in precancerous cells gives the cells a growth advantage (winners) and eliminates non-cancerous cells (losers) [10,11].

The reprogramming of glucose and glutamine metabolism is not the only key event in oncogenesis; indeed, a particular focus has been recently directed toward the involvement of lipid metabolism in supporting growth, particularly when nutrients are limited, and de novo lipogenesis and lipids are now considered active components in the signaling processes involved in cellular transformation [12]. Indeed, enzymes within the Fatty Acid (FA) synthesis pathway, such as Acetyl-coA-A Lyase (ACLY), Acetyl-CoA-A Carboxylase (ACC) and Stearoyl-CoA desaturase-1 (SCD-1) have been targets of pharmacological inhibitors in an attempt to decrease their effect on cancer cell proliferation [13,14,15,16].

In order to better understand the function of Myc in metabolism particularly for lipids, we used *Drosophila melanogaster* to perform a proteomic analysis in the fat cells to identify peptides that are predominantly induced by Myc when animals are in low nutrient conditions. Our data identified, in addition to glycolytic enzymes and components of glutamine metabolism, enzymes involved in lipid metabolism such as FAS and ACC and Desat1, a fatty acid desaturase. [7].

Stearoyl Desaturases are rate-limiting enzymes that use Stearoyl-CoA9-desaturase activity for the biosynthesis of monounsaturated fatty acids (MUFAs) into triglycerides (TGAs), phospholipids (PL) and Ceramides (CR), from stearoyl-CoA and palmitoyl-CoA [17]. In *Drosophila*, there are two genes encoding for Stearoyl Desaturases, called Desat1 and Desat2, which have 82% identity within their protein sequences and are more than 50% identical to their human homologues- Stearoyl Desaturase 5 (SCD-5) and Stearoyl Desaturase-1 (SCD-1), respectively. The function of Stearoyl Desaturases is conserved in many species and a mutation in SCD-1 results in a dramatic reduction of body fat, insulin sensitivity and reduced overall animal size [7,18].

Fatty acids are very dynamic molecules, and, in order to be mobilized and transported, they need to be cleaved by lipolysis, a process that has often been associated with autophagy [19]. Autophagy is a physiological process present in all organisms that uses catabolic processes to balance cellular energy in response to nutrient starvation and is also important for eliminating dysfunctional organelles or toxic proteins to preserve cellular homeostasis [20]. In *Drosophila*, autophagy is induced in the fat body in response to nutrient starvation where the reduction of insulin and TOR signaling triggers autophagy to allow animal survival [21].

In tumors, the role of autophagy is complex; indeed, if a basal level is necessary for quality control and to eliminate unfit tumor cells, an increase above physiological levels gives the cells a growth advantage to the point where it may function as a survival mechanism, thereby promoting tumorigenesis [22]. Autophagy can act cell-autonomously in tumors with high levels of adipose triglyceride lipase (ATGL) in the peri-tumoral area as it increases lipolysis, resulting in tumor survival [23]. However, autophagy can act also in a non-cell-autonomous manner, as it was recently shown in pancreatic ductal adenocarcinoma in humans and in *Drosophila* where the release of unknown soluble factors and amino acids, by autophagic neighboring cells, favors the growth of tumor cells [24,25].

Myc overexpression induces autophagy with a mechanism that was attributed, both in *Drosophila* and in vertebrates, to the activation of the unfolded protein response (UPR) that promotes a PERK-dependent autophagy [26,27]. Instead, in this report, we linked Myc-induced autophagy with its ability to modulate the expression of Desat1 and lipid metabolism. As we previously showed, Myc induces the expression of several components of lipid metabolism, including Desat1, whose activity was shown to be necessary for the ability of Myc to increase triglyceride levels and to control systemic growth and enhance animal survival in low nutrient conditions [7]. Desat1 was shown to be required for starvation-induced autophagy in larval fat body [28], and our data follow up on this observation to show that Myc-induced autophagy depends on the normal physiological levels of Desat1. The functional link between Myc and Desat1 to promote autophagy is conserved not only in the fat cells, but also in the epithelial cells of the larval gut and in the epithelial monolayer composing the wing imaginal disc. Moreover, we demonstrate that Desat1 is also required for Myc ability to induce growth in cells of the wing discs, suggesting another possible mechanism for the cooperation of these genes.

The proto-oncogene *c-myc* is overexpressed in the majority of human cancers [1], while increased expression of SCD-1, and to a lesser extent SCD-5, has also been found in many solid tumors and associated with poor prognosis of survival [29,30,31].

In this report, we first outlined a novel function for Desat1 in *Drosophila* necessary for Myc-induced autophagy. Second, we correlated overexpression of Myc with high levels of SCD-1 in prostate cancer, highlighting a novel cooperation between Myc and the regulation of lipid metabolism to induce autophagy as a survival/growth mechanism.

## 2. Materials and Methods

### 2.1. Fly Husbandry

*Cg-Gal4* (Bloomington Drosophila Stock Center, Bloomington, IN, USA), *UAS-mCherry-ATG8a* [32], *UAS-Desat1-RNAi* and *yw^1118^* which is used as control and referred as wild type (WT) (Vienna Drosophila Research Center, Vienna, Austria) *yw*; *UAS-HA-dMyc*, [33], *yw*; *Act5C* > *FRT-CD2-FRT* > *Gal4*; *TM3* [34], *hsp70-flippase y^122^w*; *If*; *MRKS/SM5-TM6b* (this work). Animals were raised at low density, at 25 °C on a standard molasses meal containing 6 g/L agar, 75 g/L corn flour, 60 g/L white sugar, 50 mL/L molasses, 50 g/L beaker yeast powder, along with nipagin and propionic acid (Acros Organic, Belgium, WI, USA). 

### 2.2. Proteomics

Three independent collections of fat bodies from third instar were harvested in 0.66% Rapigest (Waters Corp., Milford, MA, USA) containing dithiothreitol (6 mM), sonicated, boiled and digested to peptides with trypsin. Analysis of peptides and statistics analysis, using analysis of variance (ANOVA) and hierarchical cluster analysis, was performed at the Quantitative Proteomics Center at Columbia University as described in [7].

### 2.3. Gene Ontology Analysis

The Database for Annotation, Visualization, and Integrated Discovery Analysis (DAVID) [35,36] was used to provide Gene Ontology (GO) annotations for proteins obtained in the proteomics analysis that showed an increase of 1.3 fold or higher, and to categorize these proteins into groups with similar biological functions. The lists generated from each genotype and feeding state were then compared to each other using Microsoft Excel (Microsoft Office 2011, USA). Protein Analysis Through Evolutionary Relationships (PANTHER) [37] database tools were also used to classify the proteins according to their biological and metabolic function and to generate pie charts displaying these different functional categories.

### 2.4. Generation of Inducible Flip-Out Clones and Clonal Analysis

Females, *yw*; *UAS-mCherry-ATG8a*; *Act5C* > *FRT-CD2-FRT* > *Gal4/TM3*, were crossed with males carrying *the heat-shock Flippase y^122^w* together with the relative UAS-transgenes. Animals were laying eggs for 3 h on grape plates with yeast, and after 24 h larvae were transferred in vials with fresh food. Heat shock was performed on larvae at 48 h after egg laying (AEL) for 15–30 min at 37 °C. Larvae were dissected at 90 AEL, mounted using MOWIOL 4-88 (Sigma-Aldrich, St. Louis, MO, USA) and mCherry fluorescence was analyzed in clones from the different organs using a LEICA SP2 confocal microscope. Quantification *of-mCherry-ATG8a* fluorescence in larval imaginal discs was calculated from 5 images for every genotype at 40× magnification, keeping the acquisition parameters constant in order to compare the intensity of the fluorescent signal corresponding to mCherry-ATG8a. For each image, an area of constant dimensions and the same intensity threshold was selected, and fluorescence intensity analysis was performed using ImageJ32 software. 

### 2.5. Starvation Assay

At least 30 staged late L2 *Cg-Gal4*; *UAS-Myc* and *Cg-Gal4* control, larvae from each genotype for each replica were collected and transferred in single well plastic plates containing paper soaked with Phosphate-buffered Saline (PBS); 10 mM Na2HPO4, 2 mMKH2PO4, 2.7 mM KCl, 137 mM NaCl). These experiments were repeated three times. Statistical analysis was performed using a *z*-test.

### 2.6. Western Blot

Fat bodies from third instar larvae were collected in lysing buffer (50 mM Hepes pH 7.4, 150 mM NaCl, 1% Triton, 1mM ethtylendiaminetetraacetic acid (EDTA) with phosphatase and protease inhibitors (Roche, Branford, CT, USA). Protein concentrations were quantified using Bradford reagent (BioRad, Hercules, CA, USA). Equal amounts were resolved on 10% SDS-PAGE gels and transferred to nitrocellulose membranes. Western blotting was performed using anti-green fluorescent protein (GFP) mouse monoclonal antibody (Roche). Enhanced chemiluminescence (ECL-GE Healthcare, Amersham, MA, USA) was used for detection.

### 2.7. Lysotracker Staining

Larvae were bisected in ice cold PBS. Fat bodies were isolated from carcasses, washed in PBS, and incubated for 30 min in a solution of Lysotracker DND-99 (Thermo Fisher, Waltham, MA, USA) 1:3000 in PBS and Hoechst 33348 (Sigma-Aldrich), fats were washed and then photographed under a Zeiss LSM510 confocal microscope (Zeiss, Germany).

### 2.8. Paraffin Embedding and Immunostaining of Human Samples

Matched tumors and normal healthy tissue samples of prostatic adenocarcinoma patients were obtained from the European Institute of Oncology (Milan) Pathology Lab archive (Milan, Italy). All samples were routinely fixed in 10% neutral buffered formalin for 16 to 24 h at room temperature and embedded in paraffin. Immunohistochemical staining was performed on formalin-fixed and paraffin-embedded prostate sections (8-μm-thick). Slides were deparaffinized, subjected to antigen retrieval by heating for 2 minutes in 0.01 M sodium citrate pH 6 and washed in PBS-0.05% Triton X-100 (Sigma-Aldrich, San Luis, MO, USA). After blocking with a PBS solution containing 5% of bovine serum albumin (BSA), prostatic tissues were incubated with rabbit monoclonal anti-MYC (Y69, Abcam, Cambridge, UK) (dilution 1:300) and mouse monoclonal SCD-1 (ab19862, Abcam, Cambridge, UK) (1:50), overnight at 4 °C, followed by an anti-rabbit Alexa555 and anti-mouse Alexa488 (Thermo Fisher, Waltham, MA, USA) secondary antibodies for 1 h at room temperature. Tissues were mounted using MOWIOL and fluorescence images were acquired using a LEICA SP2 confocal microscope (Zeiss, Oberkochen, Germany). The quantification of the mean pixels corresponding to the intensity of fluorescence in the sections was determined using the histogram tool in ImageJ. Data are expressed in arbitrary units and represent the average fluorescence intensity in a fixed area and relative standard deviations were calculated using two independent patients, each including at least ten counts for each section.

## 3. Results

### 3.1. Proteomic Profile from Fat Bodies Expressing Myc

**Figure 1:**
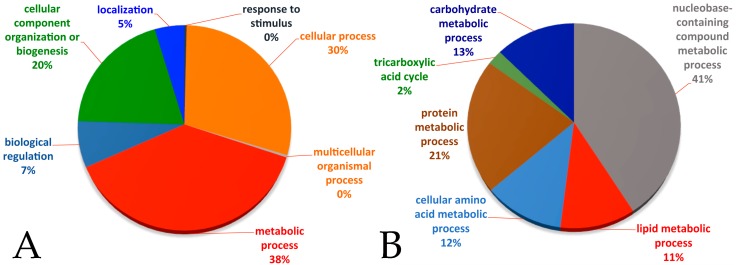
Pie chart representation of the biological (**A**) and metabolic (**B**) processes significantly changed in fat cells from animals overexpressing Myc in the fat body during starvation. Gene Ontology (GO) analysis was performed using the Database for Annotation, Visualization, and Integrated Discovery (DAVID) on the raw set of proteomics data from three sets of independent experiments.

We previously reported that Myc overexpression in the fat body activates metabolic pathways allowing for the survival of animals in low nutrient starvation [7]; this prompted us to perform a proteomic analysis to identify novel proteins that contribute to maintaining the basal level of metabolism in the fat cells when nutrients are reduced. GO analysis using DAVID of the raw proteomics data reveals significant changes in the levels of proteins in the glucose and glutamine metabolic pathways and also in components of lipid metabolism (raw data and classification are in Supplementary 1). Integrated analysis of the data shows that in Myc overexpressing fat bodies, 38% of the proteins increased are involved in metabolism (Figure 1A).

GO classification, according to the primary metabolic pathways, places lipid metabolic processes as one of the major classes that changes in Myc overexpressing cells upon starvation (Figure 1B).

Table 1 reports the list of the major proteins, including components of lipid metabolic processes that are significantly increased (Fold change > 1.3, *p* value < 0,05, all data are in the Supplementary 1) in starved versus fed fat bodies from animals overexpressing Myc.

### 3.2. Myc Increases the Expression of Autophagic Genes and Induces Autophagy in the Fat Body from Animals Under Starvation

**Figure 2:**
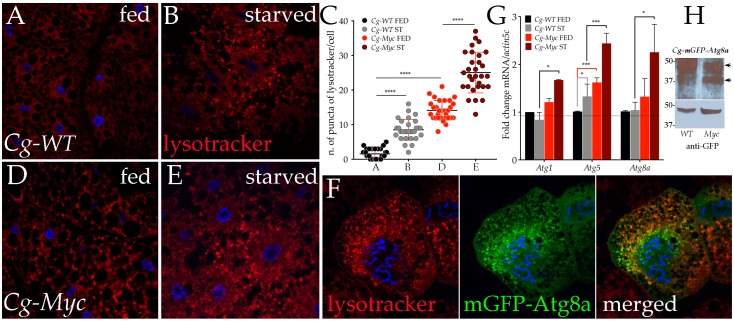
Myc expression induces autophagy in fat body from animals in nutrient starvation. (**A**–**E**) photographs (20×) of fat bodies from fed or starved animals expressing *Myc* under the control of the *Cg-Gal4* promoter. Lysotracker (red) stains the acidic vesicles, Hoechst for nuclei (blue), and (**C**) quantification of Lysotracker vesicles in fat cells; (**F**) photographs (40×) of cells expressing *Myc* together with *mGFP-ATG8a* in clones using the *actin-Gal4* promoter, left panel, Lysotracker (red), middle panel, mGFP-ATG8a (green), and right panel merged; (**G**) Quantitative real-time PCR RT-PCR of *Atg1, 5 and 8* gene expression in fat bodies from larvae of the indicated genotype; mRNA expression is normalized to *actin5C mRNA* used as control. The *p*-values in panel C and G indicates: * = *p* < 0.05, *** = *p* < 0.001, **** = *p* < 0.0001 and were calculated from Student’s *t*-test from at least three independent experiments, error bars indicate the standard deviations. (**H**) Western blot from fat bodies showing the cleavage of mGFP-ATG8a (about 50 KDa) in the indicated cell extracts is visible as a band of 37 KDa recognized by anti GFP antibody (arrows, upper panel); actin was used as a control for loading (lower panel).

Our proteomic analysis showed an enrichment, in starved cells overexpressing Myc, of proteins such as: CathepsinD (+2.31 fold, *p* = 6.07 × 10^−37^); TER94 (CG2331) (+1.41, *p* = 0); an AAA ATPase; FK506 binding protein-1 (+2.56, *p* = 2.72 × 10^−13^); Sod2 (Superoxide dismutase 2 (Mn)) (+1.94, *p* = 2.94 × 10^−21^); and Ubiquitin activating enzyme 1 (+1.22, *p* = 5.76 × 10^−18^). All proteins have been associated with the formation or maturation of the autophagosome [38,39,40], thus suggesting a function for Myc in autophagy during starvation. In order to analyze this aspect, we expressed *Myc* in the cells of the fat body using the *Cg-Gal4* promoter and quantified acidic vesicles in normal and low nutrient conditions using Lysotracker, a stain for acidic vesicles (Figure 2).

Quantification of the red-positive puncta show that while autophagy was physiologically induced upon starvation in fat cells from the control *Cg-Gal4* animals, (panels A-B quantification in C), the expression of Myc leads to a significant increase in the number of Lysotracker positive vesicles, visible already in feeding conditions (panel D) and that further increased upon starvation (panel E). Additionally, we found an induction of *Atg1*, *Atg5* and *Atg8 mRNAs* in *Myc* overexpressing fat body cells, upon starvation, while *Atg5-mRNA* was also significantly increased in the fat body of control *Cg-WT* starved animals (Figure 2G).

To avoid any detrimental effects of Myc overexpression in entire tissues and organs, we limited its expression to a small number of cells using a genetic technique where Myc was overexpressed only in clones, together with mGFP-Atg8a as marker for autophagy [41]. Using the *Actin-CD2-Gal4* flip-out cassette (see Materials and Methods), we ectopically expressed *UAS-Myc; UAS-GFP-Atg8a* in clones induced throughout the whole animal and showed co-localization of mGFP-Atg8a autophagosome vesicles with positive Lysotracker puncta in fat cells, indicating that the acidic vesicles induced by Myc were actually autophagosomes (Figure 2F). During the process of autophagy, Atg8a is cleaved by Atg4 proteases [20]; therefore, using the GFP-Atg8a fusion protein, it is possible to visualize the proteolytic process by measuring the molecular weight of free GFP cleaved from GFP-Atg8a in a Western blot [27]. Using extracts from fat bodies of *Cg-UAS-GFP-Atg8a* animals expressing *UAS-Myc* or *Cg-WT*, we showed that Myc enhances the presence of a fast migrating band that is recognized by anti GFP antibodies, indicating that Myc activates the proteolytic cleavage of Atg8a that occurs during autophagy (Figure 2H).

### 3.3. Physiological Levels of Desat1 Are Necessary for Myc-Induced Autophagy

**Figure 3:**
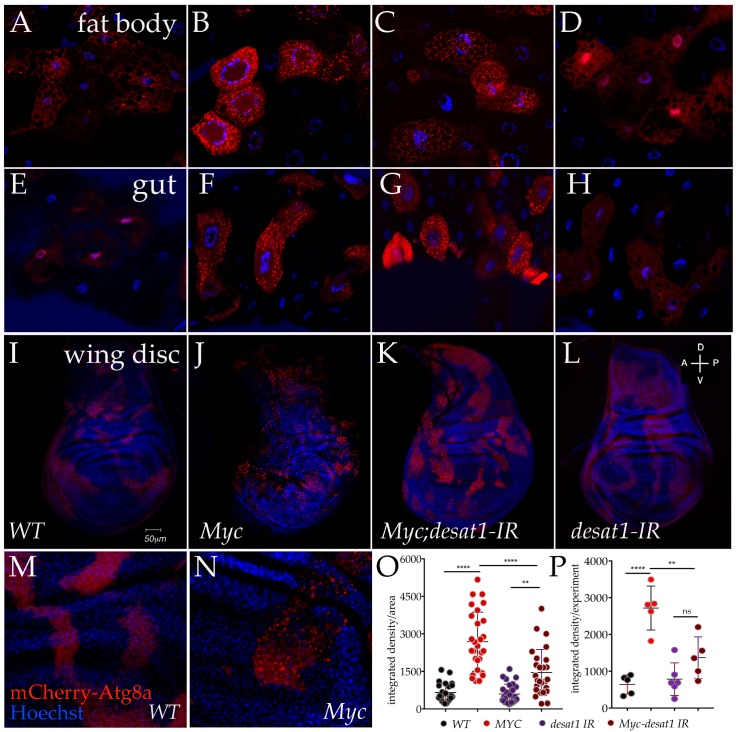
Myc induced autophagy depends on Desat1 expression. Photographs of cells from clones in the fat bodies (20×) (**A**–**D**), guts (20×) (**E**–**H**), and wing imaginal discs (10×) (**I**–**L**) expressing *UAS-mCherry-ATG8a* and the indicated transgenes from animals dissected at 80 h after egg laying (AEL). In panels M and N is show an higher magnification (20×) of control clones expressing *UAS-mAtg8a (wt)* and *UAS-mAtg8a*; *UAS-Myc*. (**O**,**P**) quantification of mCherry-Atg8a puncta in cells from the wing imaginal discs; (**O**) is representing all counts/genotype while in P data are represented as experiments/genotype. The *p*-values indicates: ** = *p* < 0.01 **** = *p* < 0.0001 and were calculated using ANOVA from at least 5 independent experiments, and error bars indicate the standard deviations.

We previously showed that functional levels of Desat1 are necessary for the systemic control of growth induced by Myc in the fat body. Desat1 was shown to be necessary for starvation-induced autophagy [28], and we therefore analyzed if its expression was a limiting factor for the function of Myc in autophagy. Using the flip-out technique, we used the *actin-CD2-Gal4* cassette to ectopically expresse *UAS-Myc* in clones and concomitantly reduced the level of Desat1 using *UAS-Desat1-RNAi*. Autophagy was analyzed by co-expression of *UAS-mCherry-Atg8a* that localizes as red vesicles in autophagosomes [32], in the fat bodies as well as in the cells from the gut and in the epithelial cells of the wing imaginal discs. As we can see in Figure 3, expression of *UAS-Myc* alone strongly induced the formation of mCherry-Atg8a positive vesicles visible in all the tissues (panels B, F and J), whereas co-expression of *UAS-Desat1-RNAi* resulted in a significant reduction of the number of vesicles (panels C, G and K), while in control cells (panels A, E and I) or in cells expressing *UAS-Desat1-RNAi* alone (panels D, H and L), the autophagic puncta were at background levels. In order to better analyze this process, we used ImageJ to quantify the autophagic vesicles in the epithelial cells of the wing imaginal discs where the puncta were more homogeneously distributed within the cells. Quantification of mCherry-Atg8a fluorescence in vesicles was expressed as integrated density measured in a fixed area of the clones (see Materials and Methods). Panels M and N show clones from the wing imaginal disc at higher magnification, used for the analysis. Data are expressed as the average of the integrated density measured in all the biological replicates enrolled in the experiments (panel O), and as the average of the experiments (panel P). Statistical analysis using ANOVA shows that decreasing the levels of Desat1 significantly reduced the number of Atg8a puncta in Myc overexpressing cells, which was significant both when data are expressed relative to all the biological replicates (panel O, *p*-value < 0.0001) and where indicated as the average of each of the five experiments (panel P, *p*-value < 0.01).

### 3.4. Reduction of Desat1 Affects the Ability of Myc to Induce Growth in Epithelial Cells

**Figure 4:**
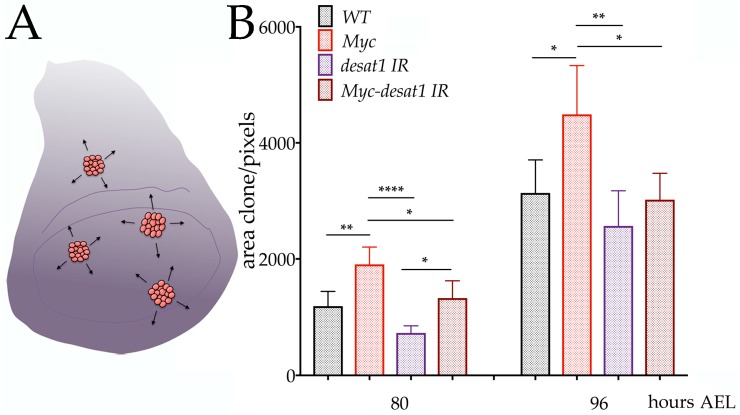
Myc induced growth depends on Desat1 expression. (**A**) schematic representation of a wing imaginal disc, clones are in red; (**B**) quantification of clonal size measured at 80 and 96 h AEL the indicated genotype. Data represents the average size of clones of the indicated genotype of four independent experiments. * = *p* < 0.05, ** = *p* < 0.01, **** = *p* < 0.001; *p*-values were calculated using one-way ANOVA with Tukey’s correction for multiple comparisons test. Error bars indicate the standard deviations within experiments.

We then analyzed whether the expression of Desat1 could modulate the ability of Myc to induce cellular growth over time. Similar to the previous experiments, we used the flip-out technique to express in clones, using the *actin-CD2-Gal4* cassette, *UAS-Myc* and *UAS-Desat1-RNAi* together with *UAS-mCherry-Atg8a* to mark the clones (Figure 4A).

Clones were induced by heat-shock at 48 h after egg laying (AEL), and their size analyzed in the wing imaginal discs from animals at 80 h and 96 h AEL. Clonal area was visualized using mCherry-Atg8a and measured from photographs; the average of the clonal area from each of the experiments is indicated in Figure 4B. Statistical analysis, using one-way ANOVA, shows that reduction of Desat1 significantly blunted the growth induced by Myc at both times of development (compare the red and brown bars) (*p*-value * < 0.05), suggesting that Desat1, thus lipid metabolism, affects Myc function to induce growth. Notably, at 80 h, reduction of Desat1 activity significantly reduced the clonal area of *UAS-Desat1-RNAi* clones (*p*-value < 0.0001) (compare the purple and brown bars); however, this effect is no longer visible at 96 h, suggesting that Desat1 may have a predominant activity earlier in development that affects cellular growth. In addition, we noted that the ability of Myc to induce growth (compare black with red bars) is less significative at 96 h. then it is at 80 h. We have done anti active-caspase3 staining and saw higher cell death in Myc clones at 96 h (data not shown), therefore we suggest that higher cell death may be responsible for the reduced clone size at the later point.

### 3.5. c-Myc and Human SCD-1 Are Co-Expressed in Epithelial Cells of Prostate Tumors

**Figure 5:**
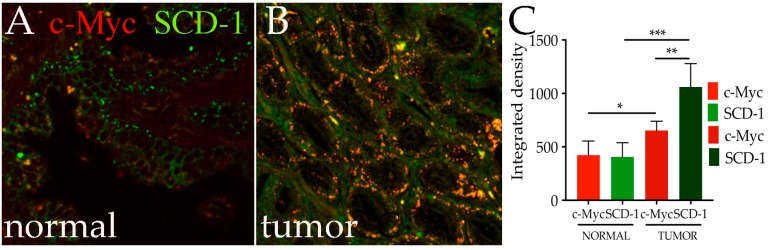
Expression of c-Myc in prostate tumors correlated with an increase in Stearoyl-CoA desaturase-1 SCD-1 protein. Photographs of normal (**A**) and prostate tumors (**B**) showing c-Myc protein expression (red) and SCD-1 (green); (**C**) quantification of c-Myc and SCD-1 protein expression expressed as integrated density in the indicated samples. * = *p* < 0.05, ** = *p* < 0.01, *** = *p* < 0.001; *p*-values were calculated from Student’s *t*-test from two independent experiments, error bars indicate standard deviations within experiments.

The growing evidence for a prominent role of lipids in tumor growth, together with the fact that human SCD-1, and more recently also SCD-5, have been found expressed and associated with the malignancy of the disease [31,42], prompted us to analyze if the expression of c-Myc correlates with increased levels of SCD-1 or SCD-5 in tumors with higher prognosis of tumorigenesis. Since SCD-1 protein is increased in prostate tumors [31], we decided to analyze whether c-Myc and SCD-1 proteins are co-expressed in histological tumor samples from patients and control tissues using immunofluorescence assays. This analysis showed that, while in non-cancerous tissue, c-Myc is expressed in the epithelial nuclei at endogenous physiological level (Figure 5A); in malignant cells, its expression is significantly increased along with the level of SCD-1 (Figure 5B). This observation is confirmed by a quantitative analysis of their levels of protein expression, which was quantified using specific antibodies against c-Myc and SCD-1 and by measuring the integrated density of the fluorescence of the two proteins spotted in the same area using ImageJ. These data show a significant increase in c-Myc levels (red) in tumor cells, as compared to normal tissues (Figure 5C, *p*-value < 0.05). This correlates with a significant increase in the same cells of SCD-1 protein (green) (*p*-value < 0.0001). Similar results were obtained for SCD-5 (not shown). These data are of particular relevance since they suggest the existence of a potential functional axis, between c-Myc and the Stearoyl-CoA desaturases (SCDs), a key enzyme in the synthesis of lipids, that act to maintain an available pool of fatty acids that may ensure the survival of tumor cells.

## 4. Discussion

Here we report a novel relationship between Myc and the Desat1 involved in the control of autophagy and sustaining growth in epithelial cells that may be conserved in tumor cells, as we show a strong correlation between the expression of c-Myc and SCD-1 in prostate cancer.

Myc function in autophagy was previously described as a response to cellular stress and dependent on the activation of the unfolded protein response UPR [27,43]; we link here instead Myc-inducted autophagy to the presence of Desat1, a key enzyme necessary for lipid anabolism. Stearoyl-CoA (Δ9-desaturases) are key enzymes that catalyze the elongation and synthesis of MUFAs to form triglycerides and phospholipids [17]. Their function is highly conserved among species; in *Drosophila*, there are two genes encoding for Desat1 and 2 that are 82% identical in their amino acid sequences and are highly homologous to their human counterparts SCD-5 and SCD-1, respectively.

The role of lipids, and particularly of MUFAs, in sustaining survival is crucial not only for normal tissue but also for cancer cells, where, in several solid malignancies such as breast, lung, prostatic, colorectal and ovarian carcinoma, an important role for SCD-1 has been shown [17]. Increased expression of SCD-1, and to a lesser extent SCD-5, has been associated with many types of cancer, including breast cancers, with a poor prognosis of survival [29,30] Along this path, Huang et al. demonstrated the pivotal role of SCD-1 in promoting cell proliferation, migration, and invasion in lung adenocarcinoma, and its significant association with poor clinical outcome [31].

A novel function of triglycerides in tumorigenesis has recently become an area of high interest, particularly because lipids have been shown to favor the survival of tumor cells through a non-autonomous mechanism, which is associated with autophagy in neighboring cells [24,25]. We are proposing that a similar mechanism may be induced by Myc, since, in our model, we show that reduction of Desat1 impairs the ability of Myc to promote cellular growth in epithelial cells of the wing discs. This suggests that components of lipid metabolism, perhaps MUFAs, may be involved in the mechanisms that Myc employs to cause growth, may be affecting the super-competitor behavior that triggers cell competition, a non-cell-autonomous phenomenon that has been characterized in *Drosophila’s* epithelium and, more recently, also in the early steps of tumor growth [10,44].

We and others, showed that, in metabolic tissues like the fat body and the liver, Myc levels increase during caloric restriction [7,45,46], suggesting a role for Myc in activating non-canonical pathways that maintain the basic metabolic rate necessary for cellular survival. However, here we showed that Myc is able to increase autophagy in animals raised in normal feeding conditions (Figure 3) and can further increase autophagy upon starvation (Figure 2). These data imply that Myc induces autophagy with a mechanism that is not controlled only by nutrients and amino acids; indeed, we were able to show that activation of the nutrient/TOR signaling pathways induced by Rheb did not significantly decrease the ability of Myc to induced autophagy in the fat body (Figure S2), perhaps following a previous observation in mammals that suggests Myc could induce autophagy via JNK/Reactive Oxygen Species (ROS) signaling [47]. In addition, it was demonstrated that Myc induces autophagy as a response to stress conditions by activation of the UPR [27,48]. With our current data, we cannot rule out if there is cross-talk between the UPR response and Myc induced autophagy “via” Desat1 and lipid accumulation; however, it will be important in the future to rule out this link to better understand Myc’s role in autophagy.

**Figure 6:**
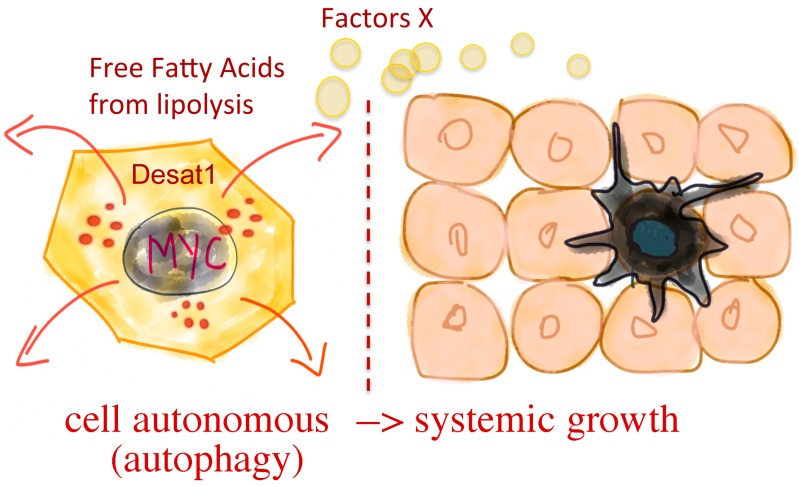
**Proposed model: potential cell autonomous and non-autonomous function of Myc in sustaining growth**. Expression of Myc favors lipid metabolism, increases the expression of Desat1 (Table 1) and autophagy (Figure 2 and Figure 3). This may result in cells that are better fit and acquire a growth advantage (Figure 4, cell-autonomously). We speculate that these cells may also secrete soluble unknown factors (Factors-X) to favor the growth of neighboring cells (non-autonomously) and even systemic growth, as we previously showed for the function of Myc and Desat1 in the fat body [7].

The data presented in this report, together with our previous observation that Desat1 is necessary for Myc to induce animal survival in starvation conditions, led us to formulate the hypothesis that the Desat1/SCD-1 axis is activated by Myc when nutrients are reduced or in specific stress conditions, in order to induce autophagy and cell survival using both a cell-autonomous mechanism that promotes de novo lipogenesis of lipophagy, and also non-autonomously by proving energy through autophagy to the neighboring cells (Figure 6).

A link between SCD-1 and autophagy has been shown in pancreatic beta cells, where inhibition of SCD-1 impaired palmitate production and functional autophagy [49]. Following our observation that links c-Myc with SCD-1 as a potential survival mechanism, we could speculate that targeting stearoyl CoA desaturase 1 as a novel therapeutic target is of particular relevance given the possibility of using SCD-1 inhibitors [50] in combination with Myc therapies to reduce tumor growth.

## 5. Conclusions

In conclusion, our data outline a novel function for Desat1 in *Drosophila* important for Myc activity in controlling autophagy and growth, which can be conserved also in humans where the cooperation between those two genes may sustain pathways “via” lipid metabolism that induce autophagy as survival mechanism in tumor cells.

## Figures and Tables

**Table 1:** Proteins components of the lipid metabolic processes enriched in the fat from starved Myc overexpressing larvae compared to starved *yw^1118^* control larvae.

UniProtKB ID	Protein Name	Fold Change	*p*-Value *	Unique Peptides **
A1Z7H2	Acyl-CoA synthetase long-chain(Acsl)	+2.74	6.41 × 10^−35^	7
ACADM	Probable medium-chain specific acyl-CoA dehydrogenase, mitochondrial	+1.71	1.48 × 10^−6^	16
MECR	Probable trans-2-enoyl-CoA reductase, mitochondrial	+1.3	1.61 × 10^−22^	3
Q9VVU1	Acyl-CoA dehydrogenase activity CG3902	+1.4	1.21 × 10^−19^	14
Q9VDT1	Arc42	+1.44	1.24 × 10^−36^	7
Q9VCC6	fatty acyl-CoA synthase activity CG6178	+1.71	3.71 × 10^−28^	7
Q9W5W8	isomerase activity CG9577	+1.34	7.82 × 10^−14^	10
Q9VXI1	3-hydroxyacyl-CoA dehydrogenase activity, CG9914	+1.5	9.76 × 10^−25^	15
CYB5R	Cytochrome b5-related(Cyt-b5-r)	+1.31	2.47 × 10^−14^	4
Q7K4Y0	Desaturase 1(Desat1)	+2.64	8.86 × 10^−43^	6
Q9BLE1	Desaturase 2(Desat2)	+1.3	1.40 × 10^−3^	2
Q7JWF1	Electron transfer flavoprotein-ubiquinone oxidoreductase(Etf-QO)	+1.64	0	9
GPDA	Glycerol 3 phosphate dehydrogenase(Gpdh)	+1.52	1.81 × 10^−39^	23
B7Z0E0	Isocitrate dehydrogenase(Idh)	+1.49	0	9
LSD1	Lipid storage droplet-1(Lsd-1)	+1.51	9.85 × 10^−13^	11
Q0KHS7	Lipid storage droplet-2(Lsd-2)	+2.77	1.87 × 10^−25^	2
Q961S9	Microsomal triacylglycerol transfer protein(Mtp)	+1.48	2.45 × 10^−5^	8
Q8IPE8	Mitochondrial trifunctional protein alpha subunit(Mtpalpha)	+1.37	1.60 × 10^−14^	22
Q9VL70	Yippee interacting protein 2(yip2)	+1.3	6.13 × 10^−64^	19

Data are from proteomic analysis and show proteins increased in Myc overexpressing cells relative to *yw^1118^* larvae with a fold change > 1.3 during starvation. * *p*-values were calculated using analysis of variance (ANOVA) test from three independent experiments; ** Unique peptides represent the repetitions of the same peptides in the proteomics analysis (see Materials and Methods).

## Supplementary Materials

The following are available online at www.mdpi.com/2073-4425/8/5/131/s1. Supplementary 1: Excel file of the raw data used in Table 1 and the results of GO analysis as PANTHER Charts used for Figure 1. Figure S2: Myc induced autophagy is TOR independent. Photographs of fat cells expressing Myc alone (panel B, GFP positive cells) or together with Rheb as an activator or TOR signaling (panel C, GFP positive cells) in animals in feeding condition, showing that Myc expression induces the formation of acidic vesicles, labeled with Lysotracker, even in the presence of Rheb, a known activator of TOR signaling. Panel A shows that expression of Rheb (GFP positive cells) inhibits autophagy induced in animals upon starvation.
